# Supplementary material for: Rein Tension in Transitions and Halts during Equestrian Dressage Training
Source: Animals (Basel). 2019 Sep 23;9(10):712. doi: 10.3390/ani9100712 (PMC6827353; doi:10.3390/ani9100712)
Supplement: Supplementary file 1 [file animals-09-00712-s001.zip › Figure S3.pdf]

Figure S3.

Boxplots of median left (red boxplot) and right rein tension (blue compact plot) per transition category and horse. Riders are numbered R1 etc. Horses within rider are labeled H1-H3. The lines show median rein tension during the sessions per gait (black=walk, green=trot, red/blue= left/right canter). The figures in the plot shows n in each box (for the right rein). Note that some data range outside the y-scaling.

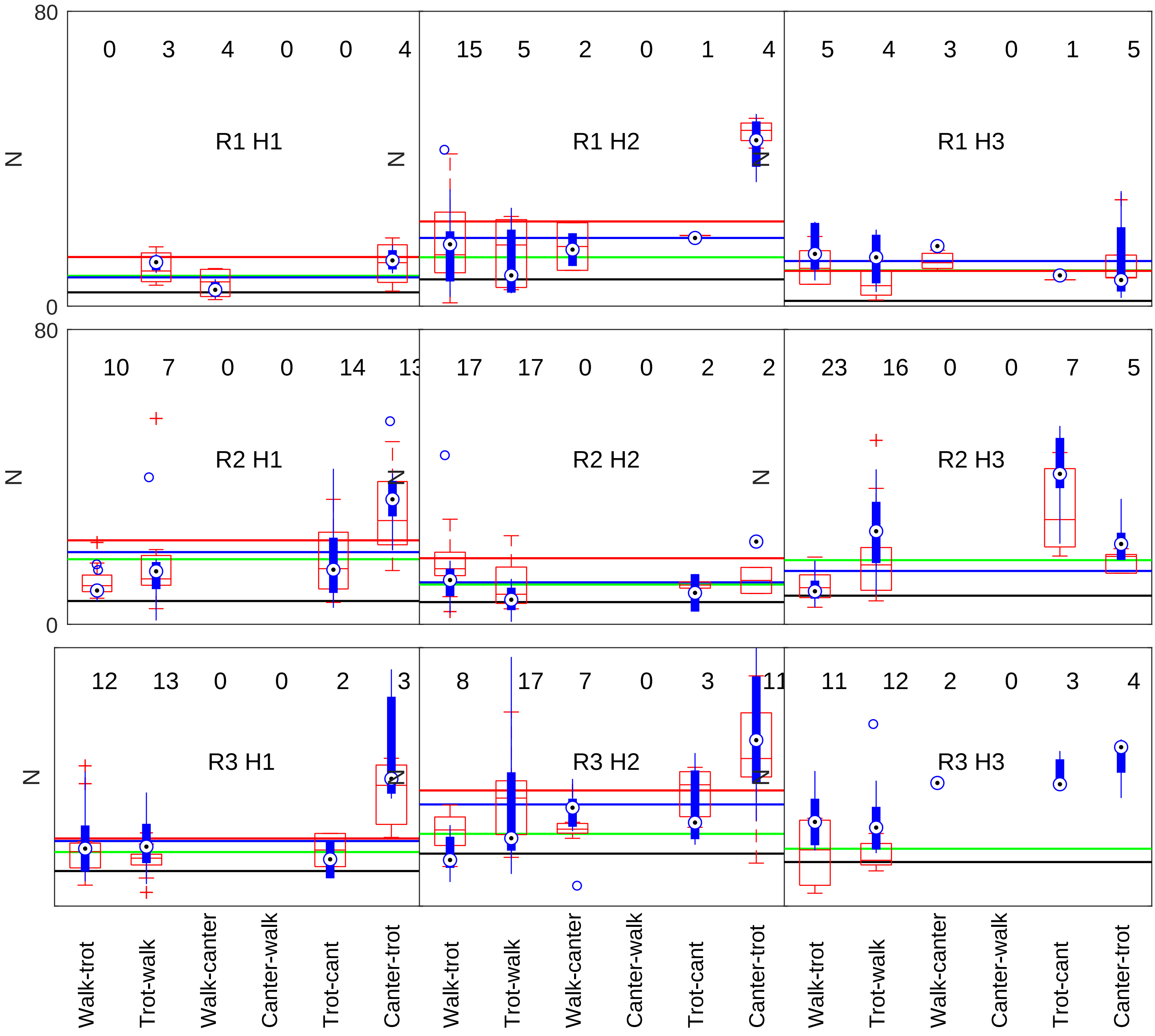
80
0
N
R1 H1
R1 H2
R1 H3
R2 H1
R2 H2
R2 H3
R3 H1
R3 H2
R3 H3
Walk-trot
Trot-walk
Walk-canter
Canter-walk
Trot-cant
Canter-trot

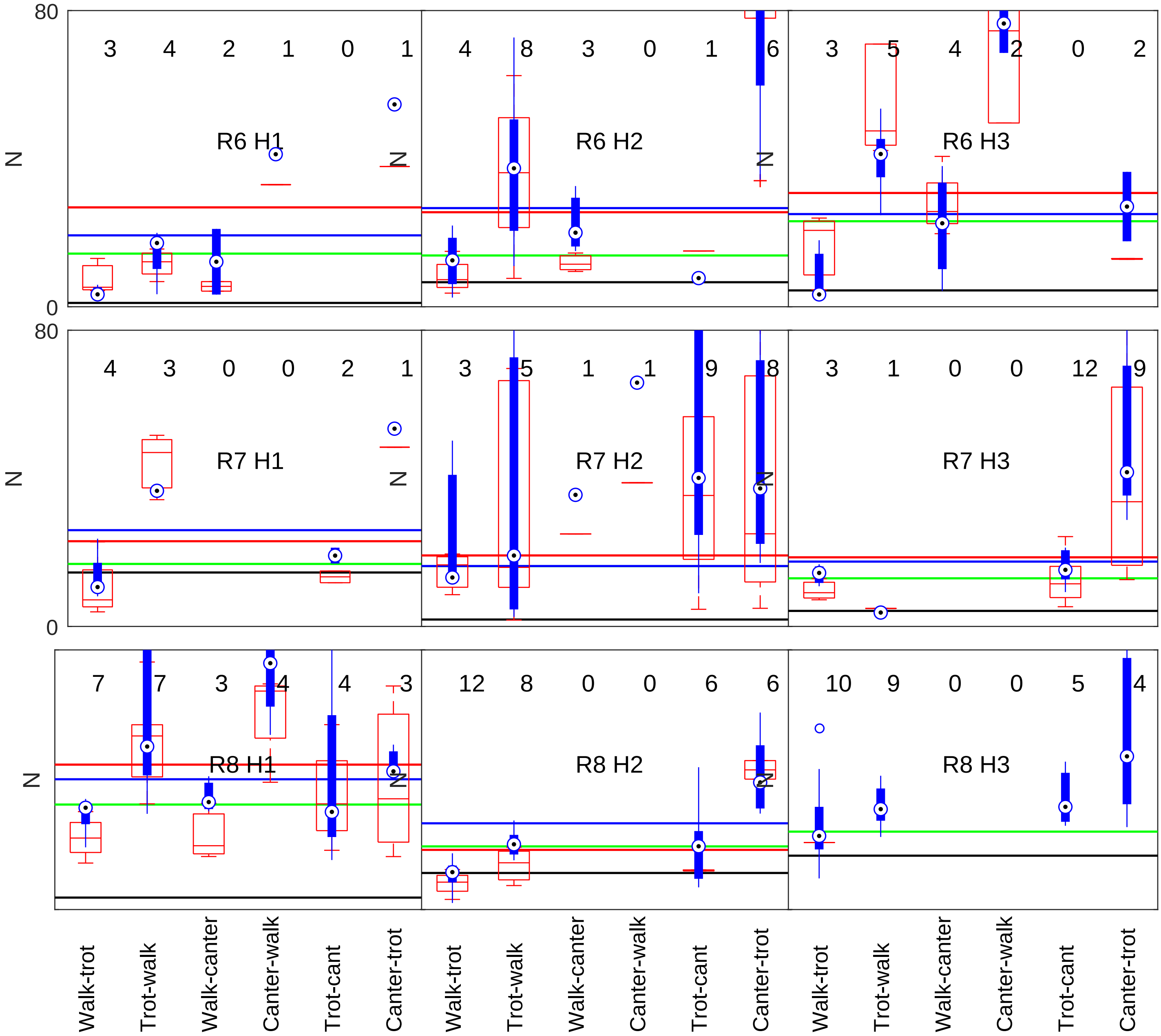
80
0
80
0
N
N
N
R6 H1
R6 H2
R6 H3
R7 H1
R7 H2
R7 H3
R8 H1
R8 H2
R8 H3
3 4 2 1 0 1
4 8 3 0 1 6
3 5 4 2 0 2
4 3 0 0 2 1
3 5 1 1 9 8
3 1 0 0 12 9
7 7 3 4 4 3
12 8 0 0 6 6
10 9 0 0 5 4
Walk-trot
Trot-walk
Walk-canter
Canter-walk
Trot-cant
Canter-trot
