# Supplementary material for: Rein Tension in Transitions and Halts during Equestrian Dressage Training
Source: Animals (Basel). 2019 Sep 23;9(10):712. doi: 10.3390/ani9100712 (PMC6827353; doi:10.3390/ani9100712)
Supplement: Supplementary file 1 [file animals-09-00712-s001.zip › SchemeS1.pdf]

Scheme S1.

Quick guide to interpretation of included angular and vertical positional data during the gait:

General background:
Inertial measurement units (IMUs) generally measure accelerations and angular velocities. In this study IMUs were positioned on the head, with the IMU fastened at the browband, and on the sacrum (x-IMU, x-io Technologies Limited, UK).
Vertical acceleration signals were double-integrated to yield velocity and vertical position data. Euler angles were derived from the x-IMU software. For integration, acceleration data were high-pass filtered with cutoff values from 0.2 to 0.5 Hz and order 1. Only relative translations can be studied, i.e. relative vertical position between peaks and valleys (it is for example not possible to study the height above ground). This quick guide is made by a combination of results from the literature and interpretation of the data (using data from Rider 8 Horse 1 for illustrations) from the current study.

Definitions:
Positive **croup roll** rotation was defined as clockwise rotation around the longitudinal axis when viewed from behind, i.e. a positive value indicated a larger degree of rotation towards the right side. Positive **nose angle**/head pitch rotation was defined as clockwise rotation around the longitudinal axis when viewed from the left side, i.e. a more positive value indicated the nose was more forward and the bridge of the nose became more horizontal. Lines in the figures below are: vertical positions of the poll (blue) and croup (magenta), croup roll (black, positive to the right when viewed from behind) and nose angle (red, positive upwards).

Trot:

The trot has been studied extensively especially with regard to lameness in horses. It is a symmetrical diagonal gait that normally has suspension phases between diagonal stance phases. The **vertical positions of the head and croup** are lowest at midstance, when the forelimb cannon bones are approximately vertical to the ground, and highest at push-off, when the horse enters the suspension phase. Regarding **croup roll**, the pelvis is in a neutral position at midstance and rolls towards the grounded hind limb through late stance then roll back to a neutral position during the first half of the contralateral hind limb stance. The **nose angle** rotates through only a small range of motion.

When horses are ridden, as in the current study, they may experience 'disturbances' from the rider that disrupt the basic gait-related pattern of nose angle and vertical head position. The nose angle and vertical head position are also easily altered by extraneous circumstances that disturb the horse's attention.

Figure for trot:

The blue horizontal bars indicate two strides starting and ending at midstance of a left hind limb

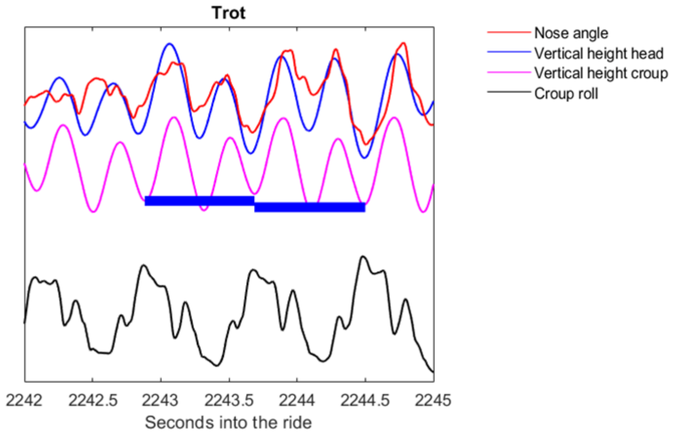

Walk:

Walk is a symmetrical four-beat gait with alternating tripedal and bipedal overlaps. There is no suspension phase. Because of the time-separation of fore and hind limbs, each limb is in midstance at a different time. The walk is associated with both pitching trunk movements and lateral trunk excursions. At forelimb midstance, the withers are supported by the forelimb reaching its relatively highest position, while the poll and croup are at their lowest vertical position. Therefore, the neck and trunk rotate in opposite directions. The **vertical head position is lowest** at forelimb midstance when the horse's trunk is rotated backwards. At the same time, the **croup vertical position is lowest,** at hind limb maximal retraction and toe-off, when the horse enters into diagonal support. **The vertical head position is highest** and the withers vertical position are lowest at dual forelimb support and coincides with hind limb midstance. The **croup vertical position is highest** at hind limb midstance. **Croup roll** reverses between forelimb and diagonal hind limb midstance, i.e. shortly after hind limb toe off, and rotates towards the hind limb in swing as in trot (the relative components of croup roll and yaw vary somewhat between horses, data not shown). Vertical head position and nose angle tend to follow each other closely and have coincident timing of the maxima and minima.

Figure for walk:

The bars indicate two strides starting and ending at left forelimb midstance.

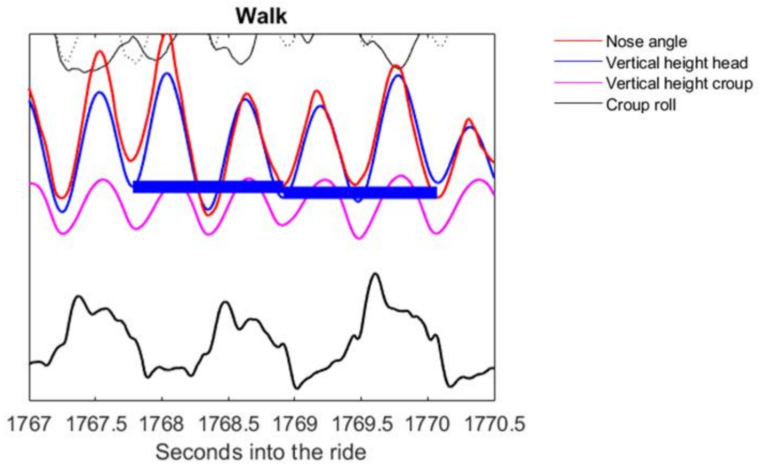

Canter:

Canter is a three-beat or sometimes four-beat gait in which the first beat is contact of the trailing hind limb, the second beat is the leading hind and trailing fore which may be slightly dissociated, and the third beat is the leading forelimb. The limbs leave the ground in the same order they were placed. The leading forelimb pushes off into a suspension phase. The **lowest vertical position of the head** will be at midstance of the leading fore (the same time as the vertical head acceleration is highest). The **highest vertical head position** is around first contact of the trailing hind. The **croup abruptly rolls** away from the leading hind limb from early stance to midstance, then rolls back towards the leading hind limb as it pushes off and as the trailing hind swings forward. This is close in time to the minimum vertical position of the head. Through the longitudinal rocking motion, the **nose angle** mimics the pattern of the vertical position of the head being smallest/largest when the vertical position of the head is lowest/highest. In the figures below, data on both left and right canter are included for the same horse and opposite croup roll motions are clearly visible.

Left/right lead canter (same horse). Each blue horizontal bar represents one stride starting and ending at midstance of left/right leading forelimb.

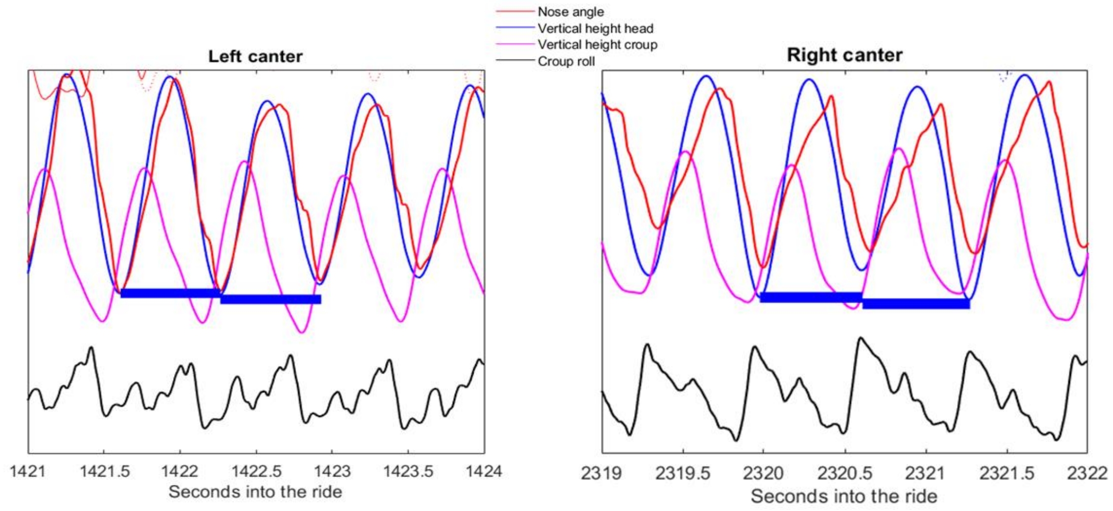

Further reading:

Buchner, H.H.F., Savelberg, H.H., Schamhardt, H.C., Barneveld, A., 1996. Head and trunk movement adaptations in horses with experimentally induced fore- or hindlimb lameness. Equine Vet. J. 28, 71–76.

Buchner, H.H., Obermuller, S., Scheidl, M. (2000). Body centre of mass movement in the sound horse. The Veterinary Journal 160, 225-234.

Goff, L., Van Weeren, P. R., Jeffcott, L., Condie, P., McGowan, C., 2010. Quantification of equine sacral and iliac motion during gait: A comparison between motion capture with skin-mounted and bone-fixated sensors. Equine Veterinary Journal 42, 468-474.

Olsen, E. Haubro Andersen, P., Pfau, T. 2012. Accuracy and Precision of Equine Gait Event Detection during Walking with Limb and Trunk Mounted Inertial Sensors. Sensors (Basel) 12: 8145–8156.

Pfau, T., Witte, T.H., Wilson, A.M. 2006. Centre of mass movement and mechanical energy fluctuation during gallop locomotion in the Thoroughbred racehorse. Journal of Experimental Biology 209: 3742-3757; doi: 10.1242/jeb.02439

Starke, S.D., Witte, T.H., May, S.A., Pfau, T. 2012. Accuracy and precision of hind limb foot contact timings of horses determined using a pelvis-mounted inertial measurement unit.
J Biomech. 45:1522-1528. doi: 10.1016/j.jbiomech.2012.03.014.
