# Supplementary material for: Rein Tension in Transitions and Halts during Equestrian Dressage Training
Source: Animals (Basel). 2019 Sep 23;9(10):712. doi: 10.3390/ani9100712 (PMC6827353; doi:10.3390/ani9100712)
Supplement: Supplementary file 1 [file animals-09-00712-s001.zip › Supplement1_r1h3_r4h2_r7h3.pdf]

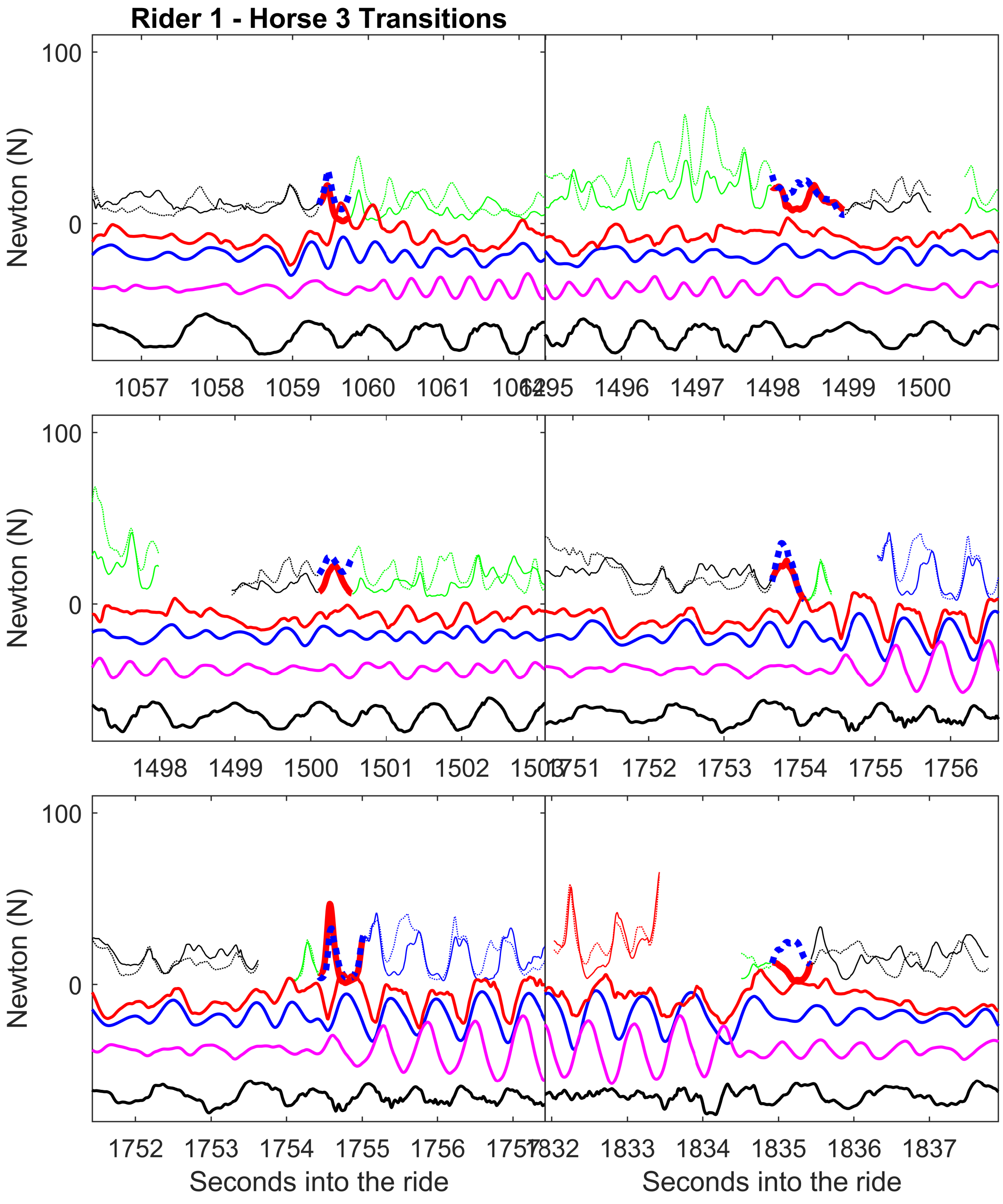
Rider 1 - Horse 3 Transitions
Newton (N)
100
0
1057
1058
1059
1060
1061
1062
1495
1496
1497
1498
1499
1500
1498
1499
1500
1501
1502
1503
1751
1752
1753
1754
1755
1756
1752
1753
1754
1755
1756
1757
1832
1833
1834
1835
1836
1837
Seconds into the ride

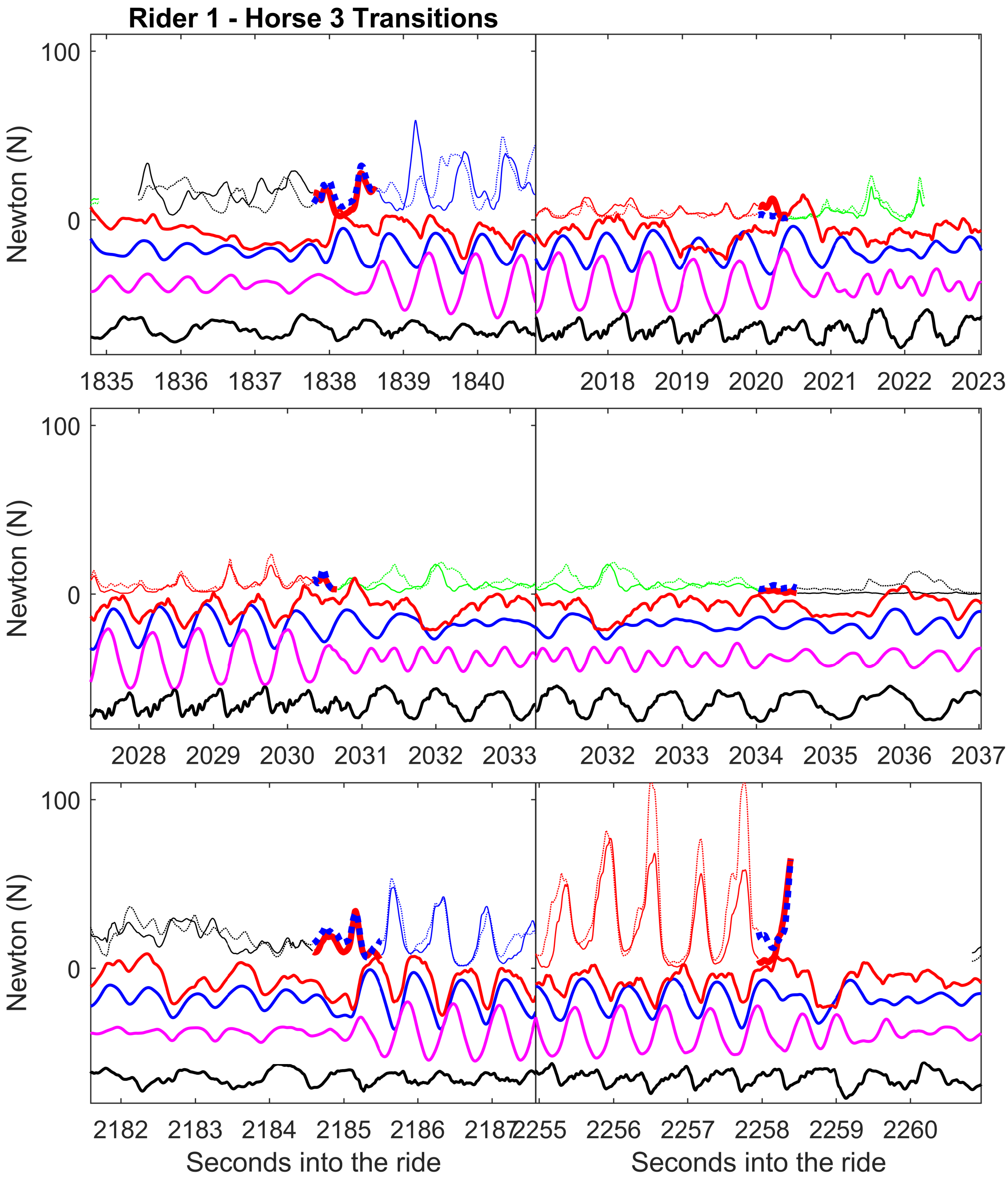
Rider 1 - Horse 3 Transitions
Newton (N)
100
0
1835 1836 1837 1838 1839 1840
2018 2019 2020 2021 2022 2023
2028 2029 2030 2031 2032 2033
2032 2033 2034 2035 2036 2037
2182 2183 2184 2185 2186 2187
2255 2256 2257 2258 2259 2260
Seconds into the ride

Rider 1 - Horse 3 Transitions

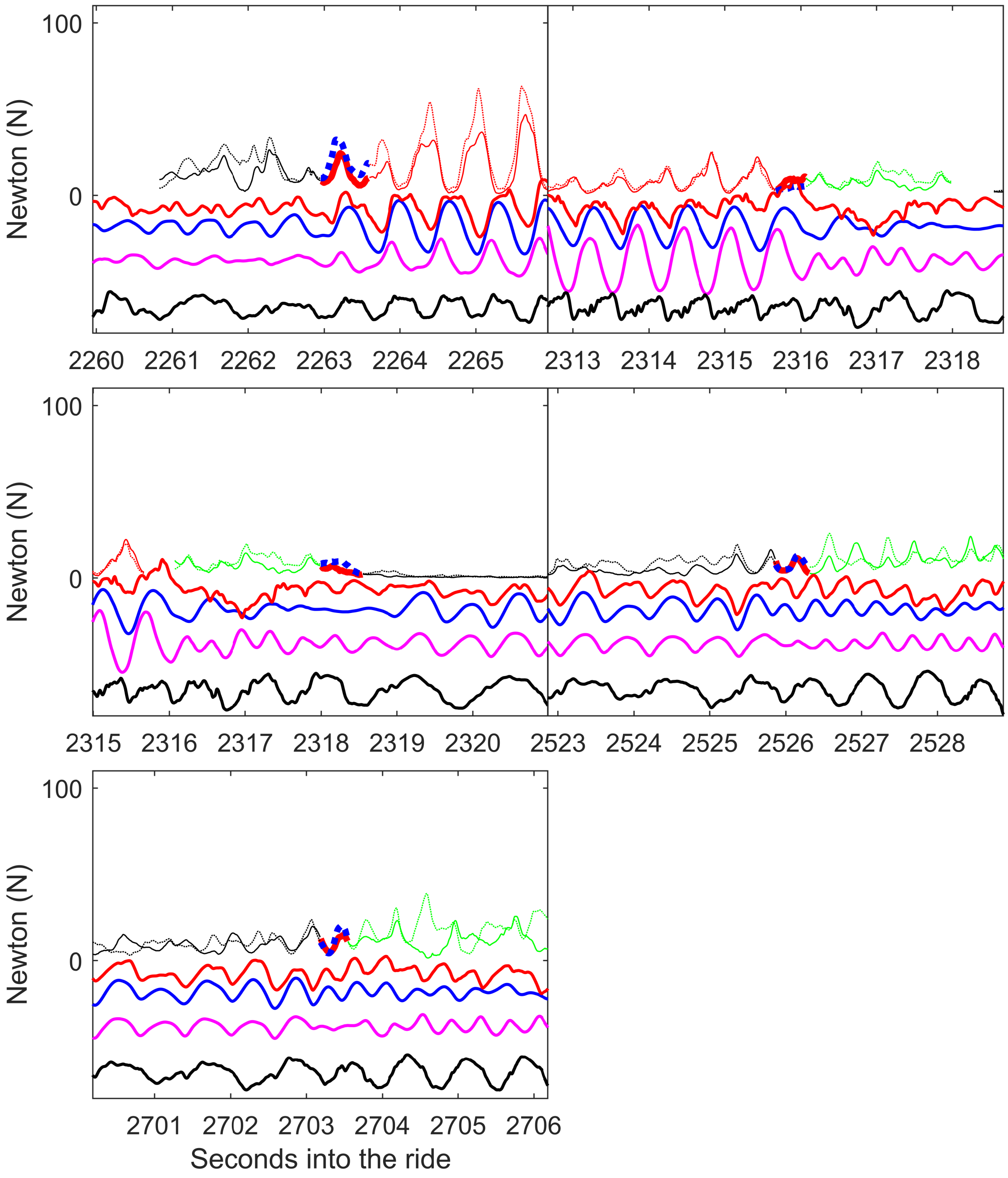

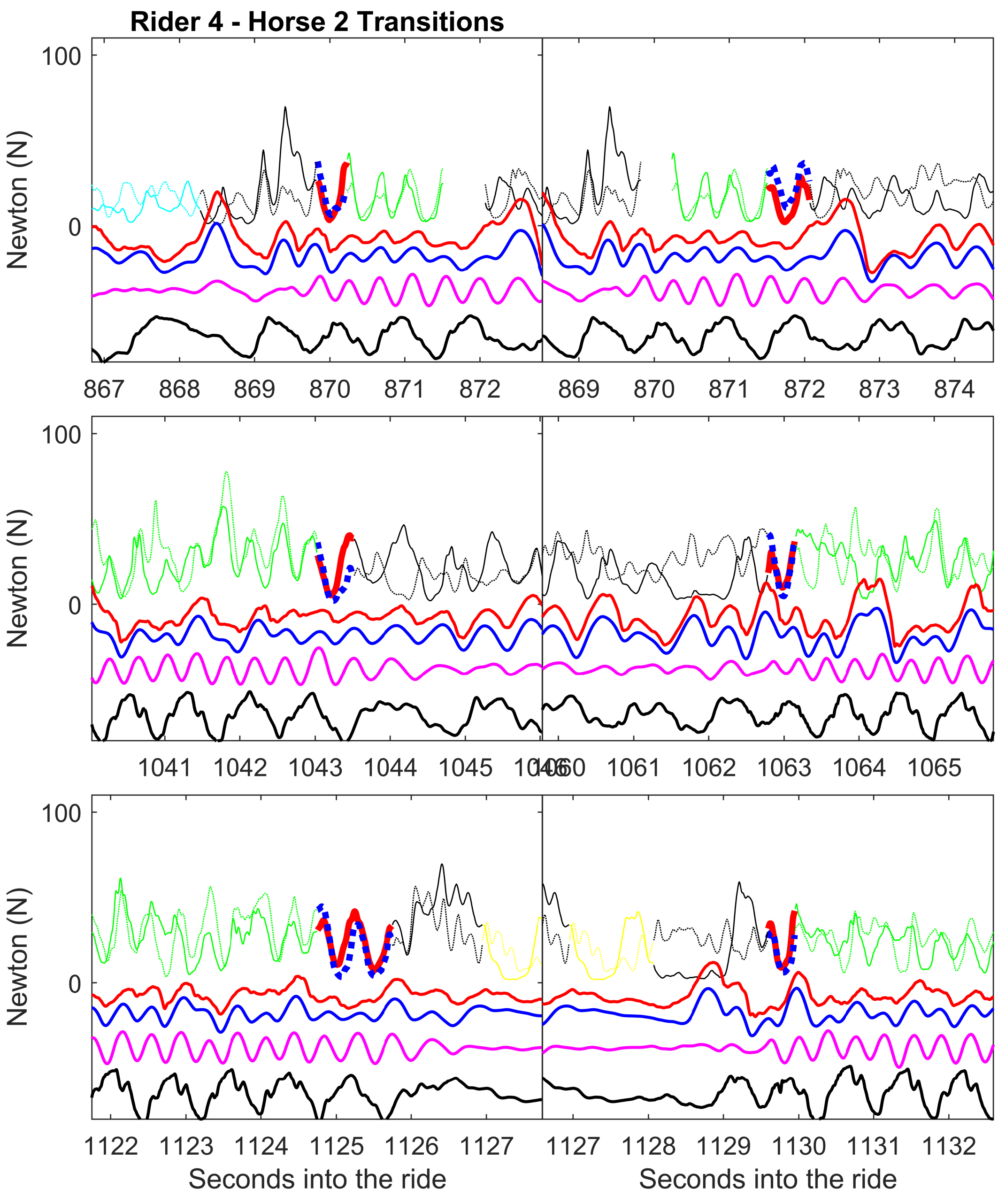
Rider 4 - Horse 2 Transitions
Newton (N)
100
0
867
868
869
870
871
872
873
874
1041
1042
1043
1044
1045
1060
1061
1062
1063
1064
1065
1122
1123
1124
1125
1126
1127
1128
1129
1130
1131
1132
Seconds into the ride

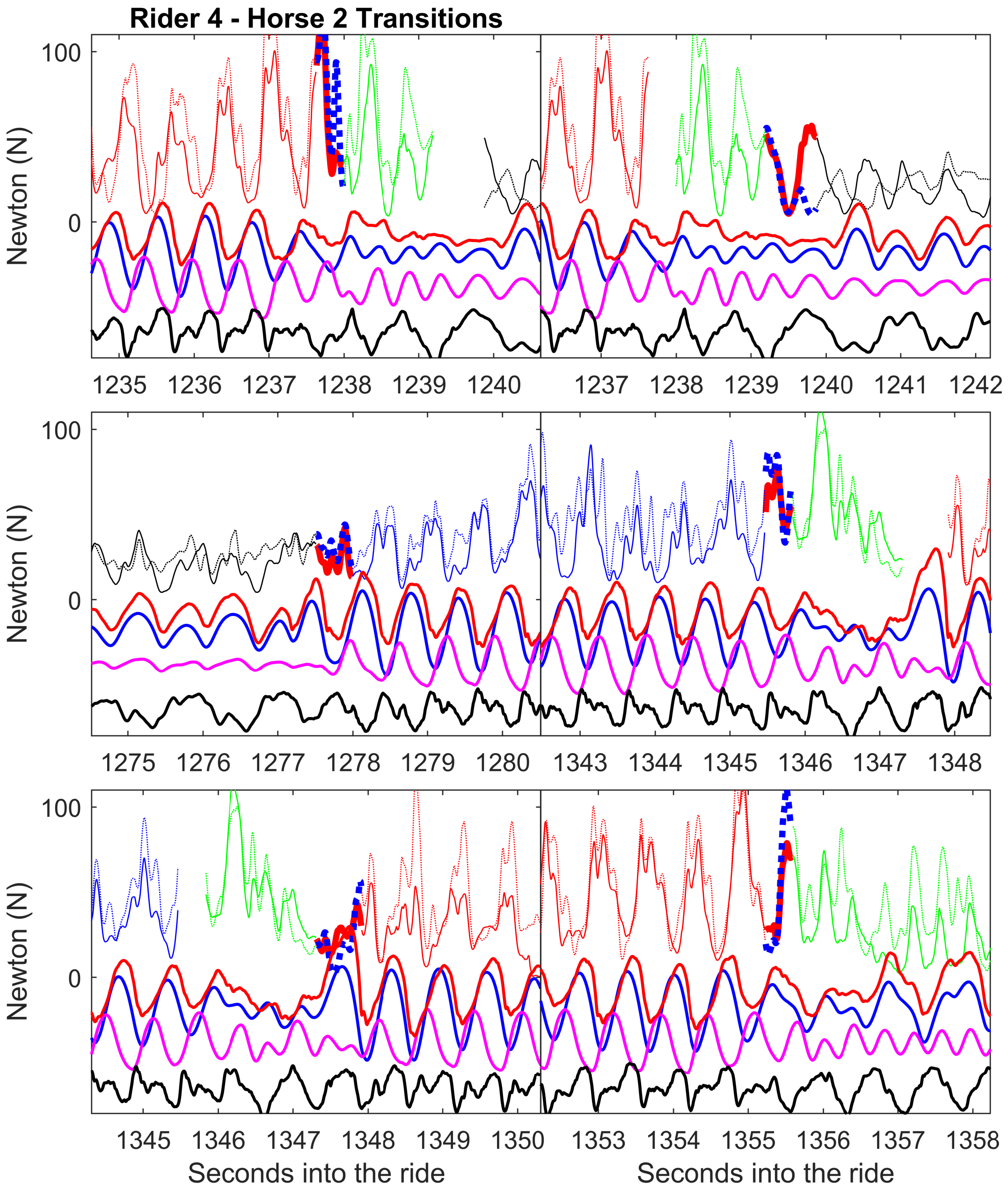
Rider 4 - Horse 2 Transitions
Newton (N)
100
0
1235
1236
1237
1238
1239
1240
1237
1238
1239
1240
1241
1242
1275
1276
1277
1278
1279
1280
1343
1344
1345
1346
1347
1348
1345
1346
1347
1348
1349
1350
1353
1354
1355
1356
1357
1358
Seconds into the ride

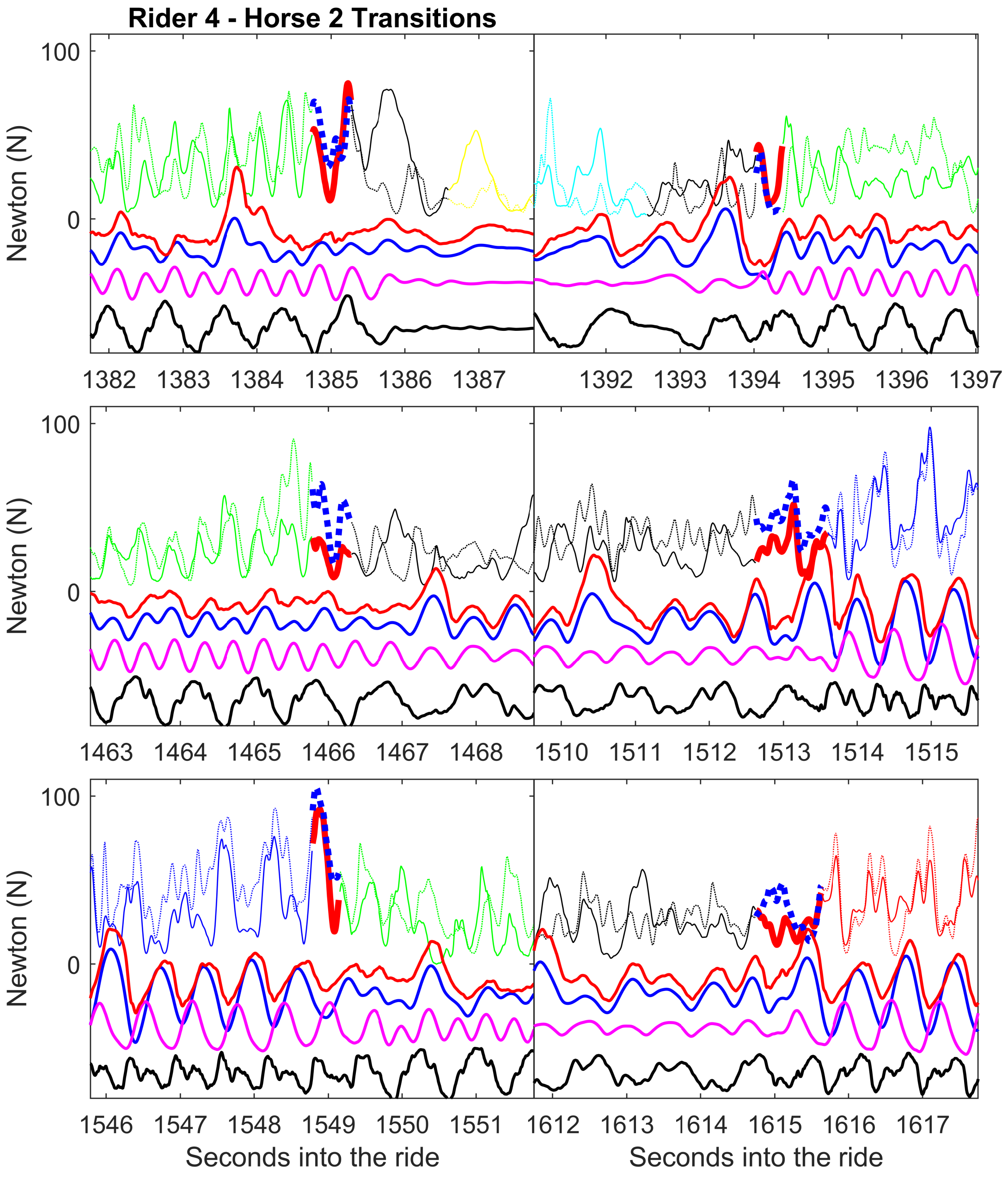

Rider 4 - Horse 2 Transitions
Newton (N)
100
0
1382
1383
1384
1385
1386
1387
1392
1393
1394
1395
1396
1397
1463
1464
1465
1466
1467
1468
1510
1511
1512
1513
1514
1515
1546
1547
1548
1549
1550
1551
1612
1613
1614
1615
1616
1617
Seconds into the ride

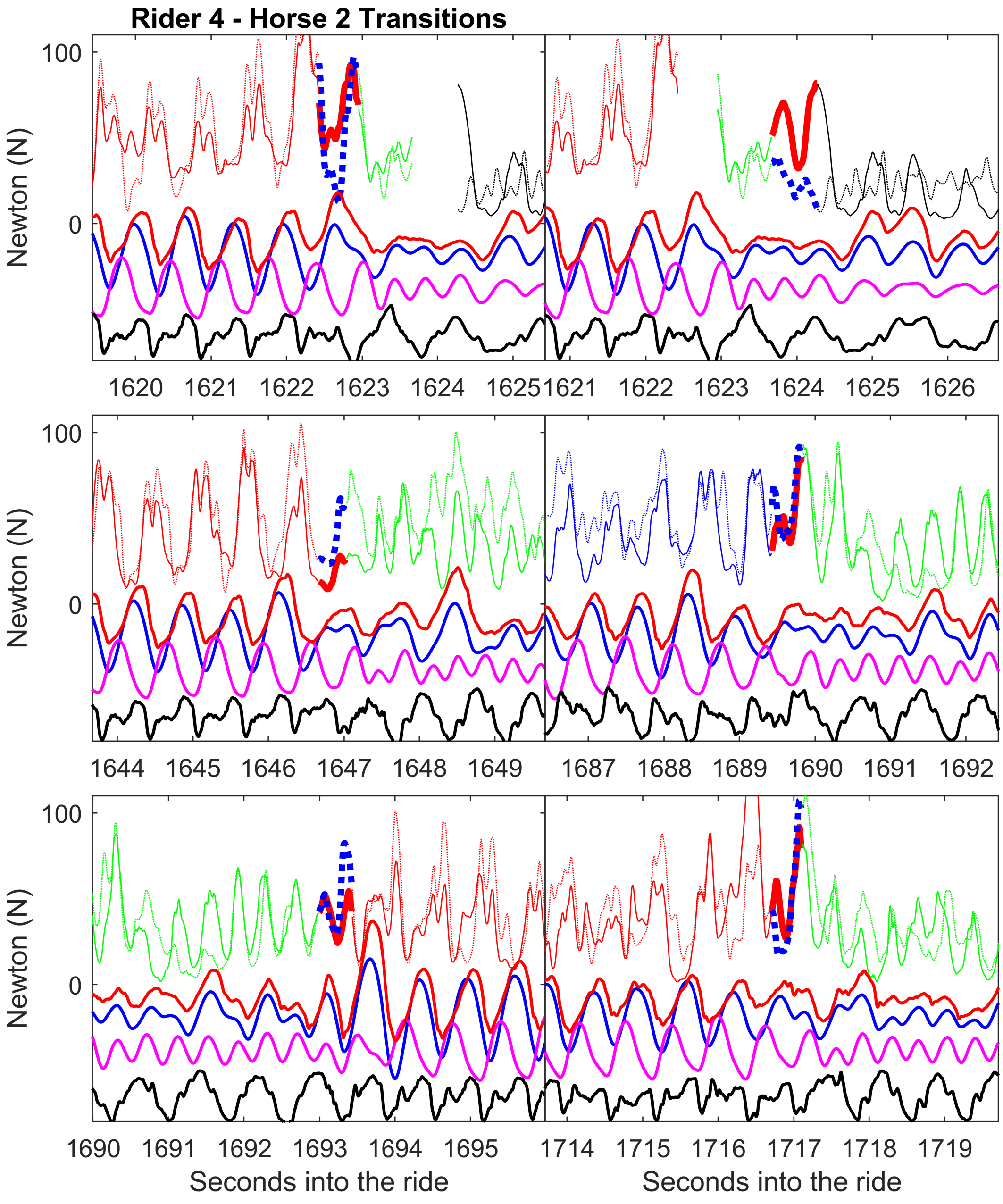

Rider 4 - Horse 2 Transitions
Newton (N)
100
0
1620
1621
1622
1623
1624
1625
1621
1622
1623
1624
1625
1626
1644
1645
1646
1647
1648
1649
1687
1688
1689
1690
1691
1692
1690
1691
1692
1693
1694
1695
1714
1715
1716
1717
1718
1719
Seconds into the ride

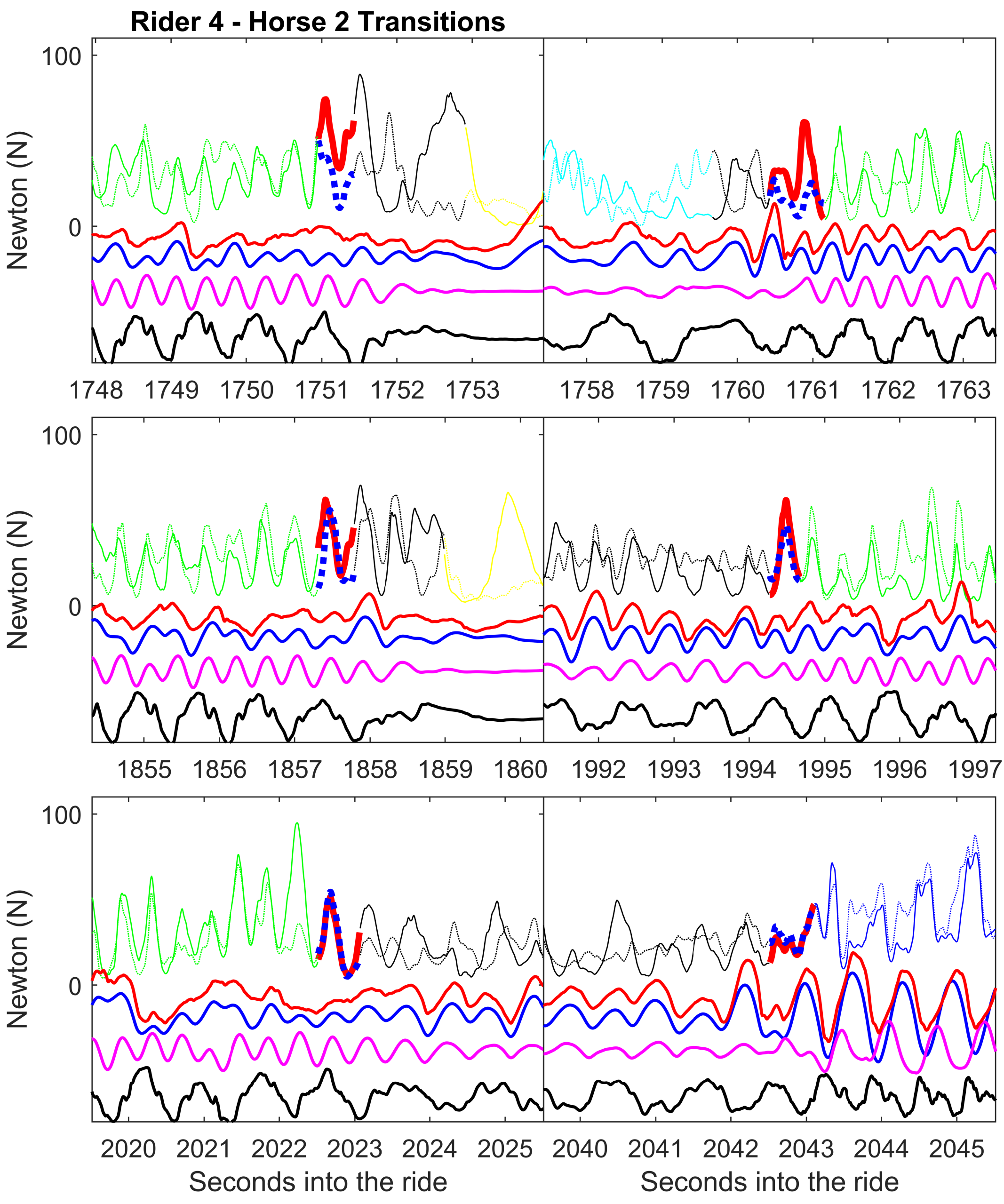
Rider 4 - Horse 2 Transitions
Newton (N)
100
0
1748
1749
1750
1751
1752
1753
1758
1759
1760
1761
1762
1763
1855
1856
1857
1858
1859
1860
1992
1993
1994
1995
1996
1997
2020
2021
2022
2023
2024
2025
2040
2041
2042
2043
2044
2045
Seconds into the ride

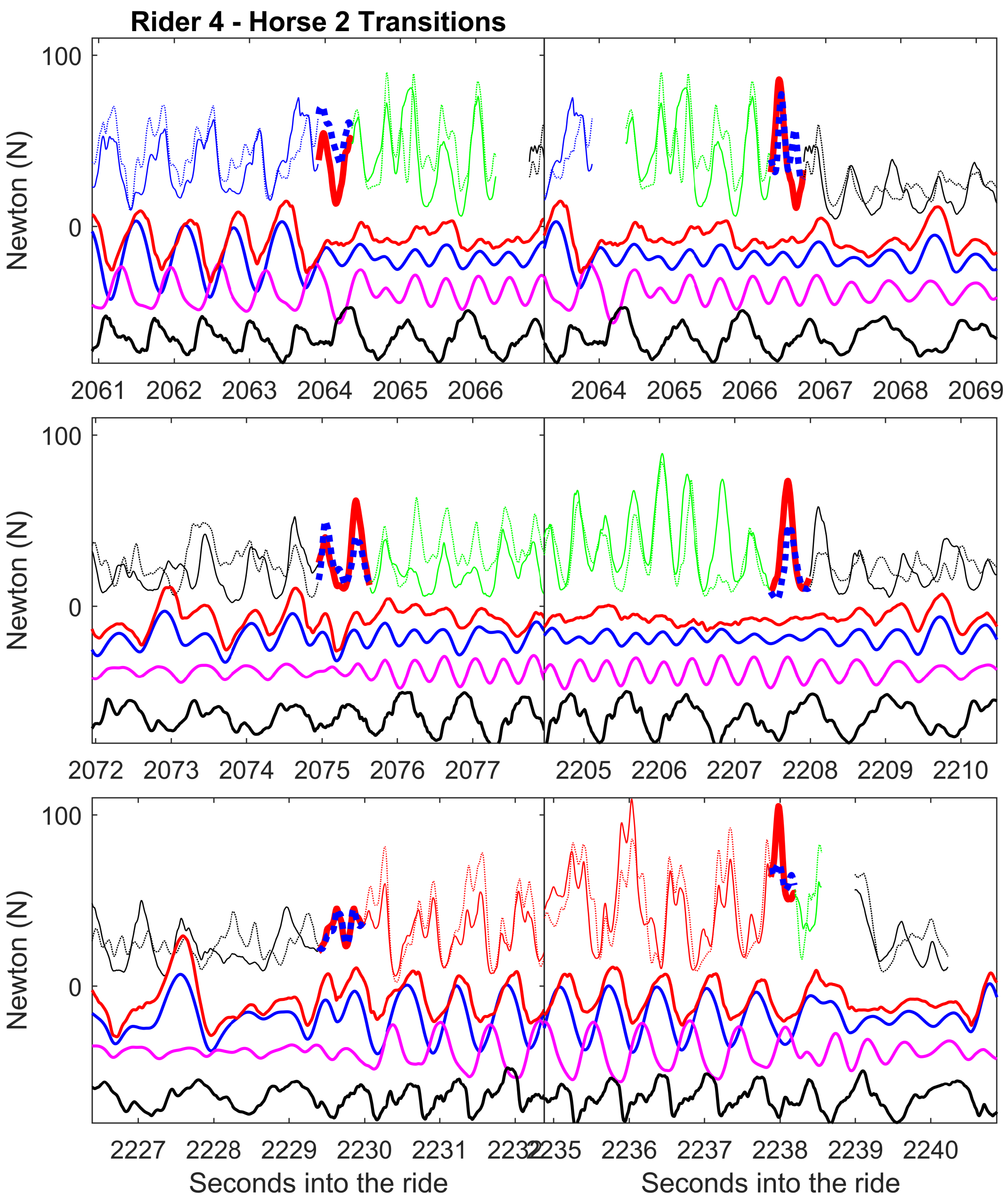

Rider 4 - Horse 2 Transitions
Newton (N)
100
0
2061 2062 2063 2064 2065 2066
2064 2065 2066 2067 2068 2069
2072 2073 2074 2075 2076 2077
2205 2206 2207 2208 2209 2210
2227 2228 2229 2230 2231 2232
2235 2236 2237 2238 2239 2240
Seconds into the ride
Seconds into the ride

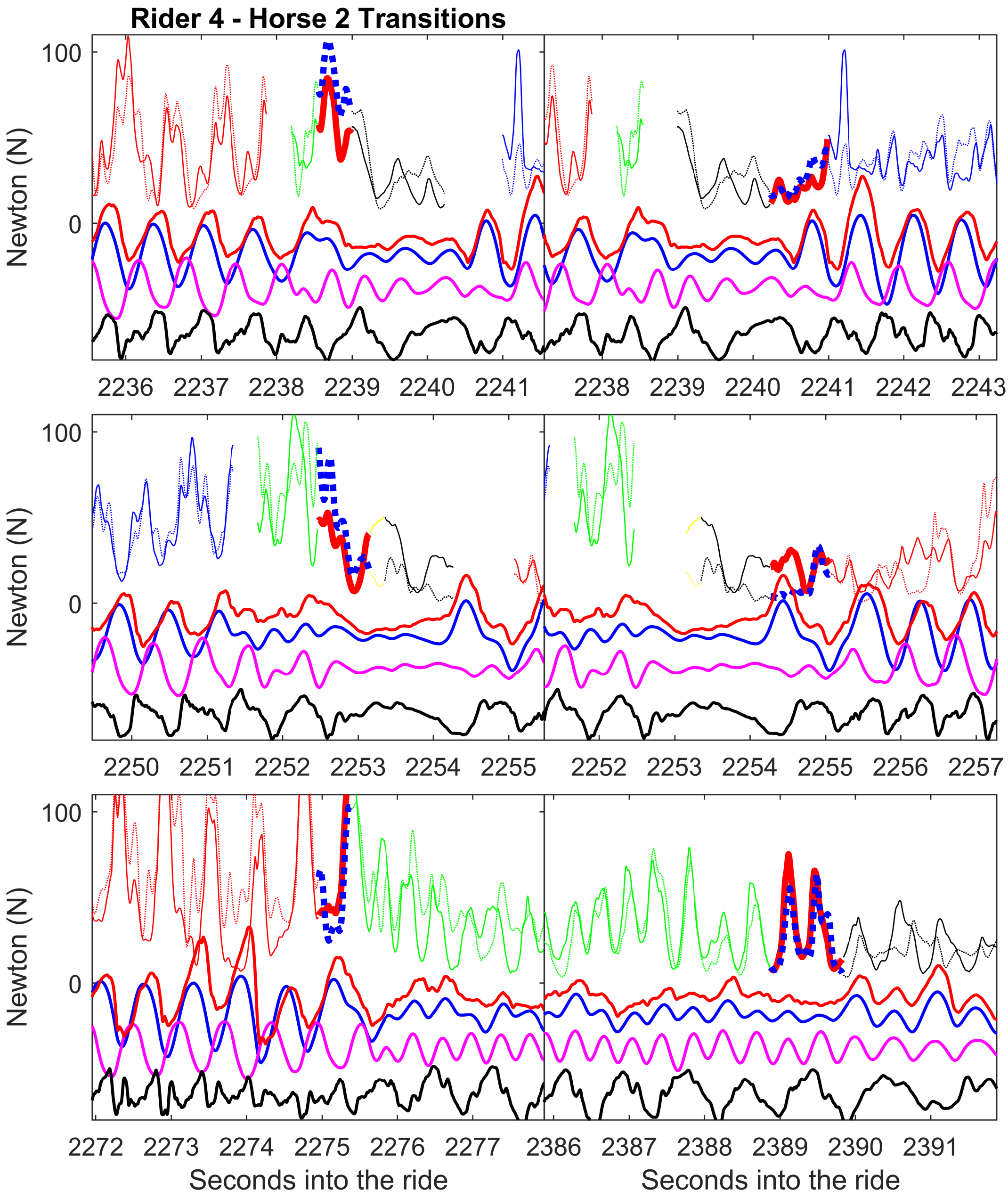

Rider 4 - Horse 2 Transitions
Newton (N)
100
0
2236
2237
2238
2239
2240
2241
2238
2239
2240
2241
2242
2243
2250
2251
2252
2253
2254
2255
2252
2253
2254
2255
2256
2257
2272
2273
2274
2275
2276
2277
2386
2387
2388
2389
2390
2391
Seconds into the ride

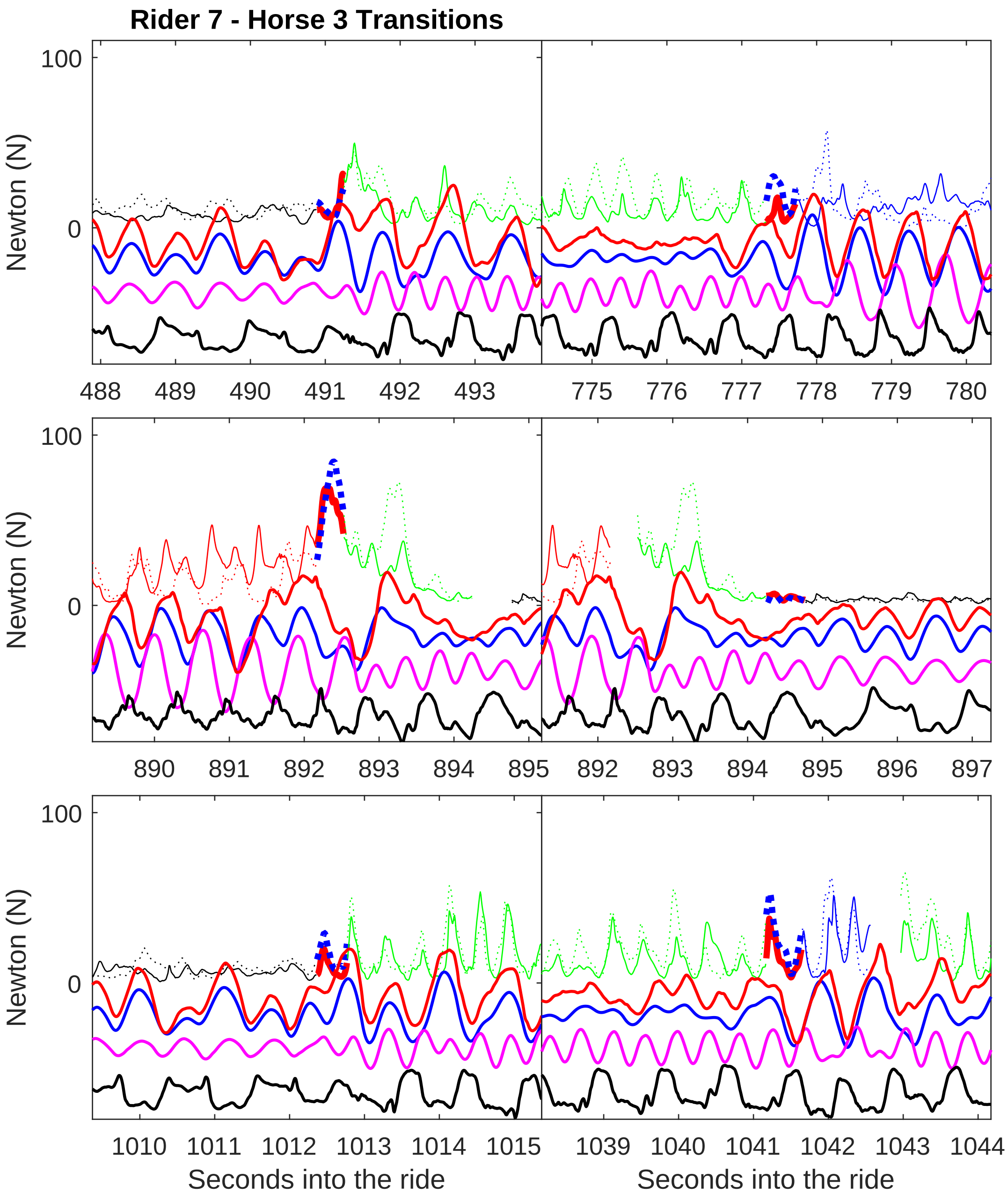
Rider 7 - Horse 3 Transitions
Newton (N)
100
0
488
489
490
491
492
493
775
776
777
778
779
780
890
891
892
893
894
895
892
893
894
895
896
897
1010
1011
1012
1013
1014
1015
1039
1040
1041
1042
1043
1044
Seconds into the ride
Seconds into the ride

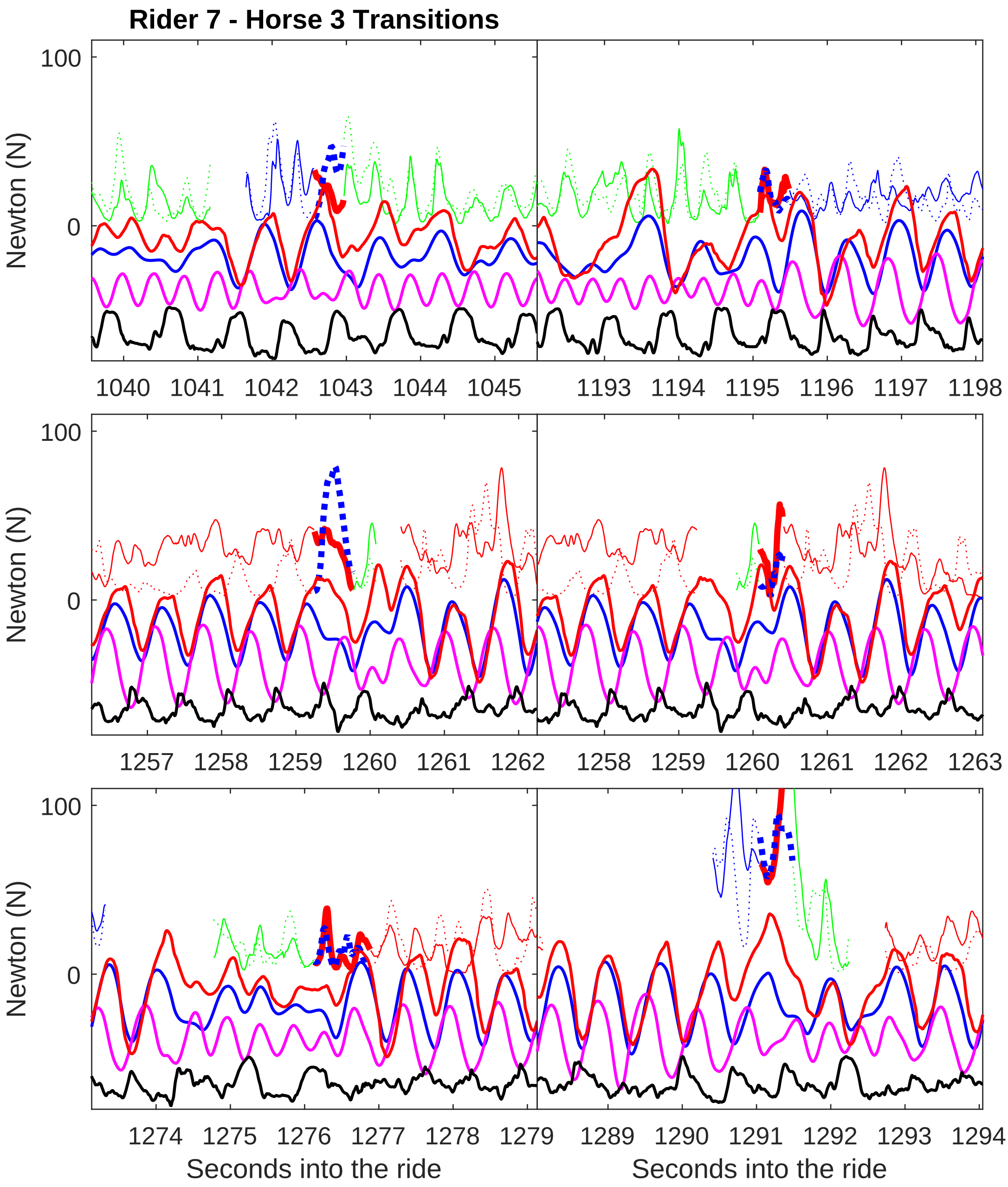
Rider 7 - Horse 3 Transitions
100
0
Newton (N)
1040
1041
1042
1043
1044
1045
1193
1194
1195
1196
1197
1198
100
0
Newton (N)
1257
1258
1259
1260
1261
1262
1258
1259
1260
1261
1262
1263
100
0
Newton (N)
1274
1275
1276
1277
1278
1279
1289
1290
1291
1292
1293
1294
Seconds into the ride
Seconds into the ride

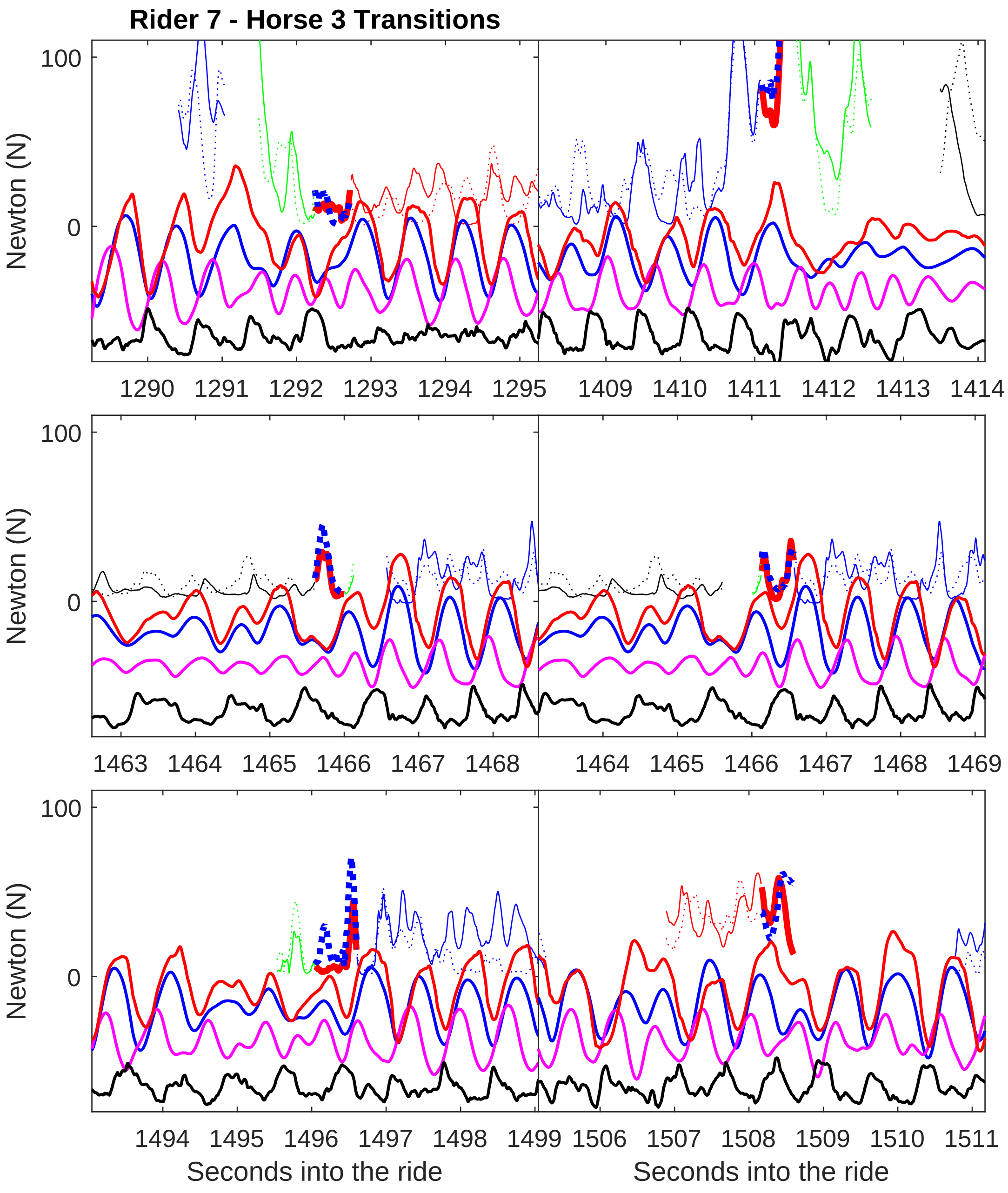

Rider 7 - Horse 3 Transitions
Newton (N)
100
0
1290 1291 1292 1293 1294 1295
1409 1410 1411 1412 1413 1414
1463 1464 1465 1466 1467 1468
1464 1465 1466 1467 1468 1469
1494 1495 1496 1497 1498 1499
1506 1507 1508 1509 1510 1511
Seconds into the ride

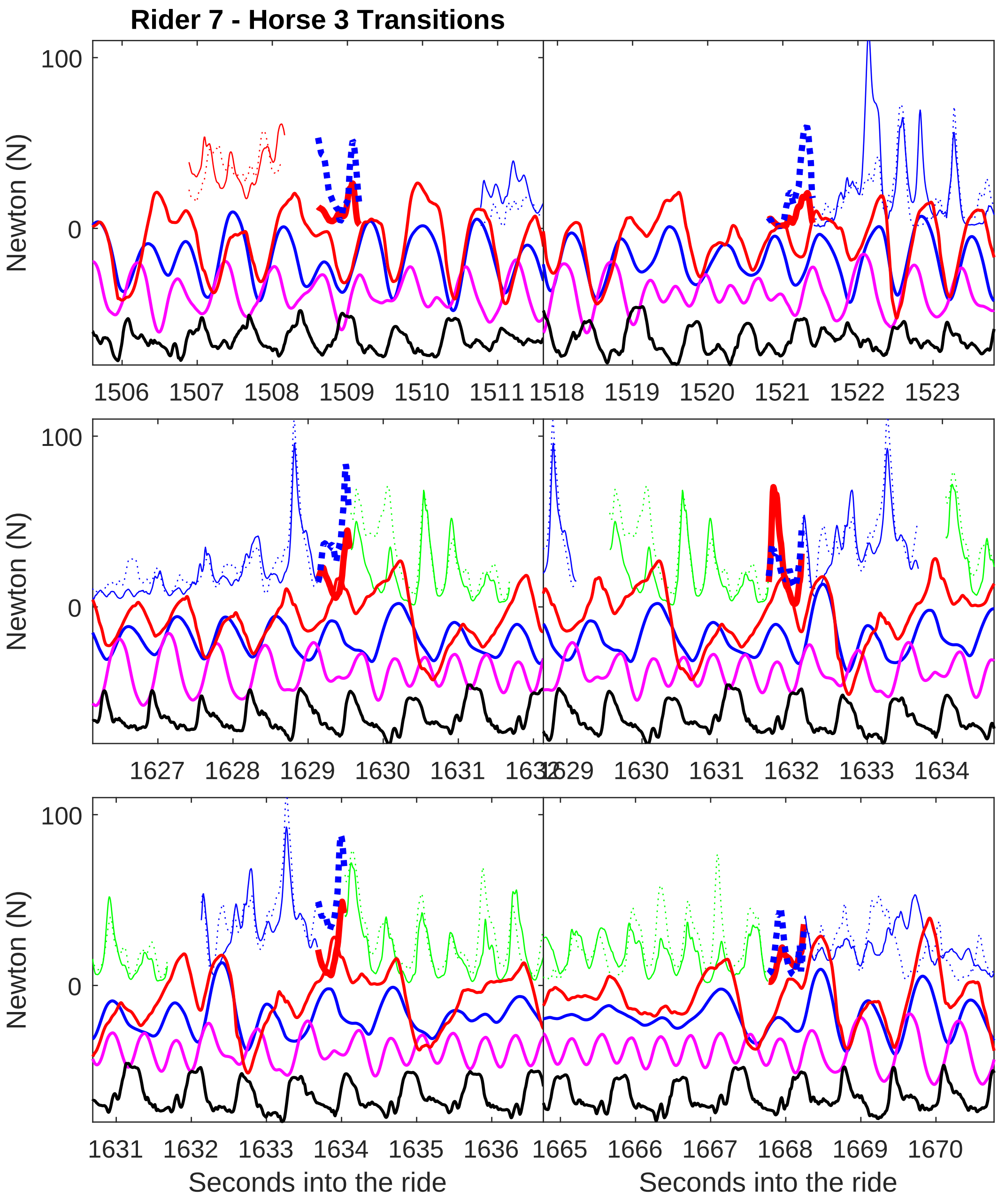

Rider 7 - Horse 3 Transitions
100
0
Newton (N)
1506
1507
1508
1509
1510
1511
1518
1519
1520
1521
1522
1523
1627
1628
1629
1630
1631
1632
1629
1630
1631
1632
1633
1634
1631
1632
1633
1634
1635
1636
1665
1666
1667
1668
1669
1670
Seconds into the ride
Seconds into the ride

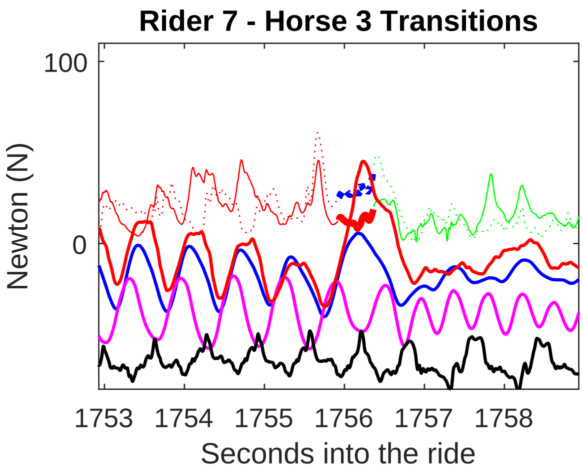
Rider 7 - Horse 3 Transitions
100
0
Newton (N)
1753
1754
1755
1756
1757
1758
Seconds into the ride
