# Supplementary material for: Rein Tension in Transitions and Halts during Equestrian Dressage Training
Source: Animals (Basel). 2019 Sep 23;9(10):712. doi: 10.3390/ani9100712 (PMC6827353; doi:10.3390/ani9100712)
Supplement: Supplementary file 1 [file animals-09-00712-s001.zip › Supplement2_r1h2_r4h3_r7h3.pdf]

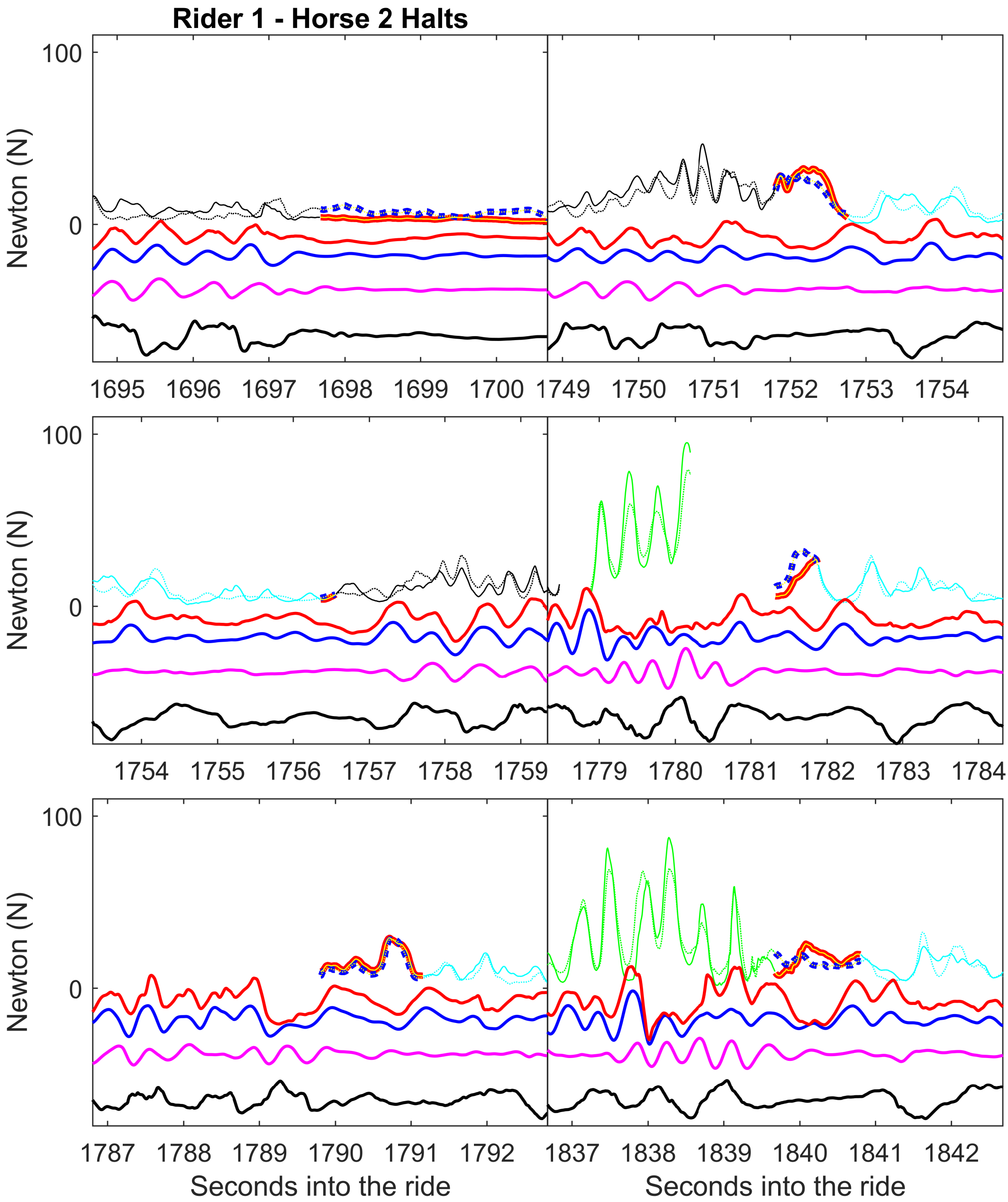

Rider 1 - Horse 2 Halts
Newton (N)
100
0
1695
1696
1697
1698
1699
1700
1749
1750
1751
1752
1753
1754
1755
1756
1757
1758
1759
1779
1780
1781
1782
1783
1784
1787
1788
1789
1790
1791
1792
1837
1838
1839
1840
1841
1842
Seconds into the ride

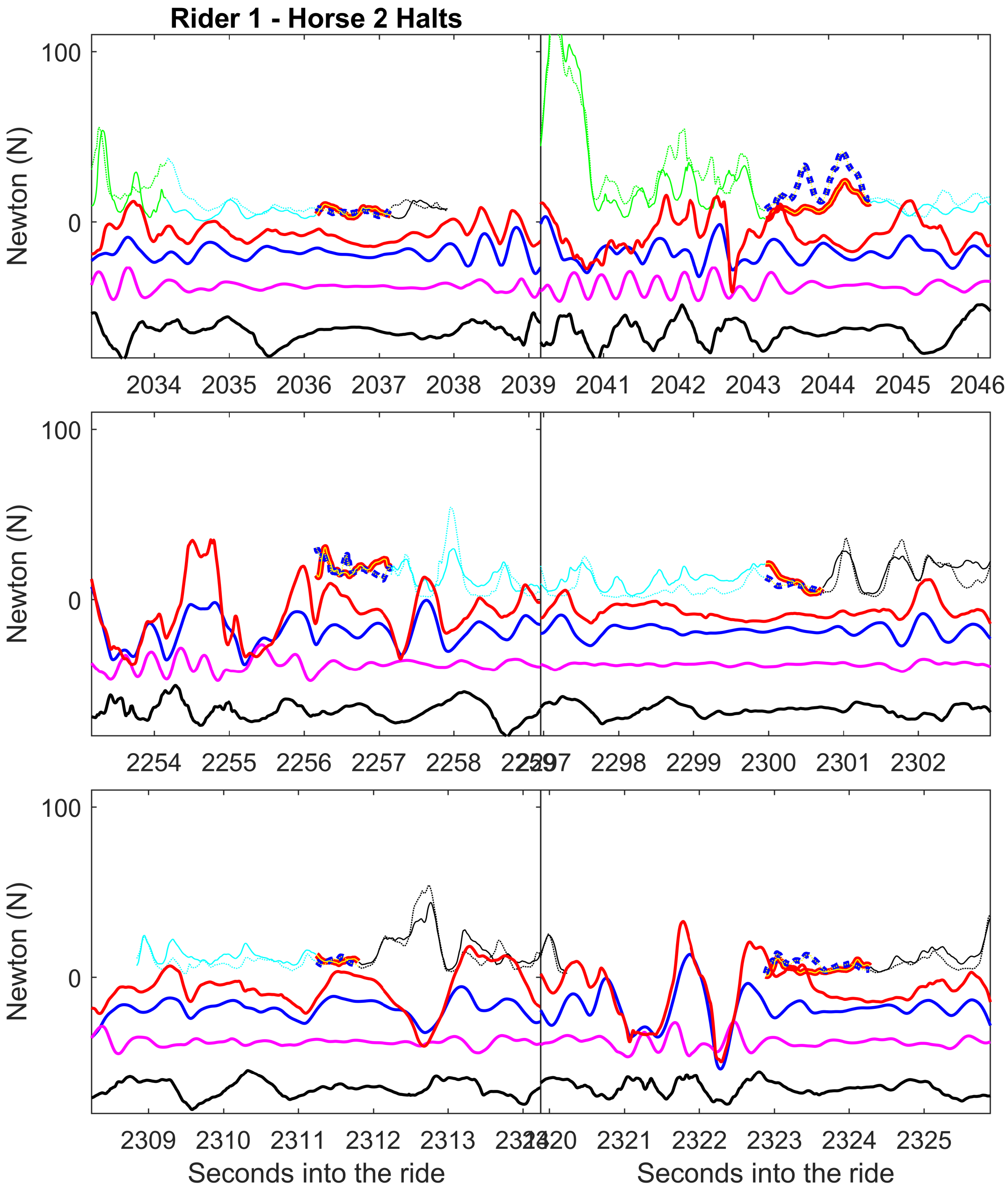

Rider 1 - Horse 2 Halts
Newton (N)
100
0
2034
2035
2036
2037
2038
2039
2041
2042
2043
2044
2045
2046
2254
2255
2256
2257
2258
2298
2299
2300
2301
2302
2309
2310
2311
2312
2313
2321
2322
2323
2324
2325
Seconds into the ride
Seconds into the ride

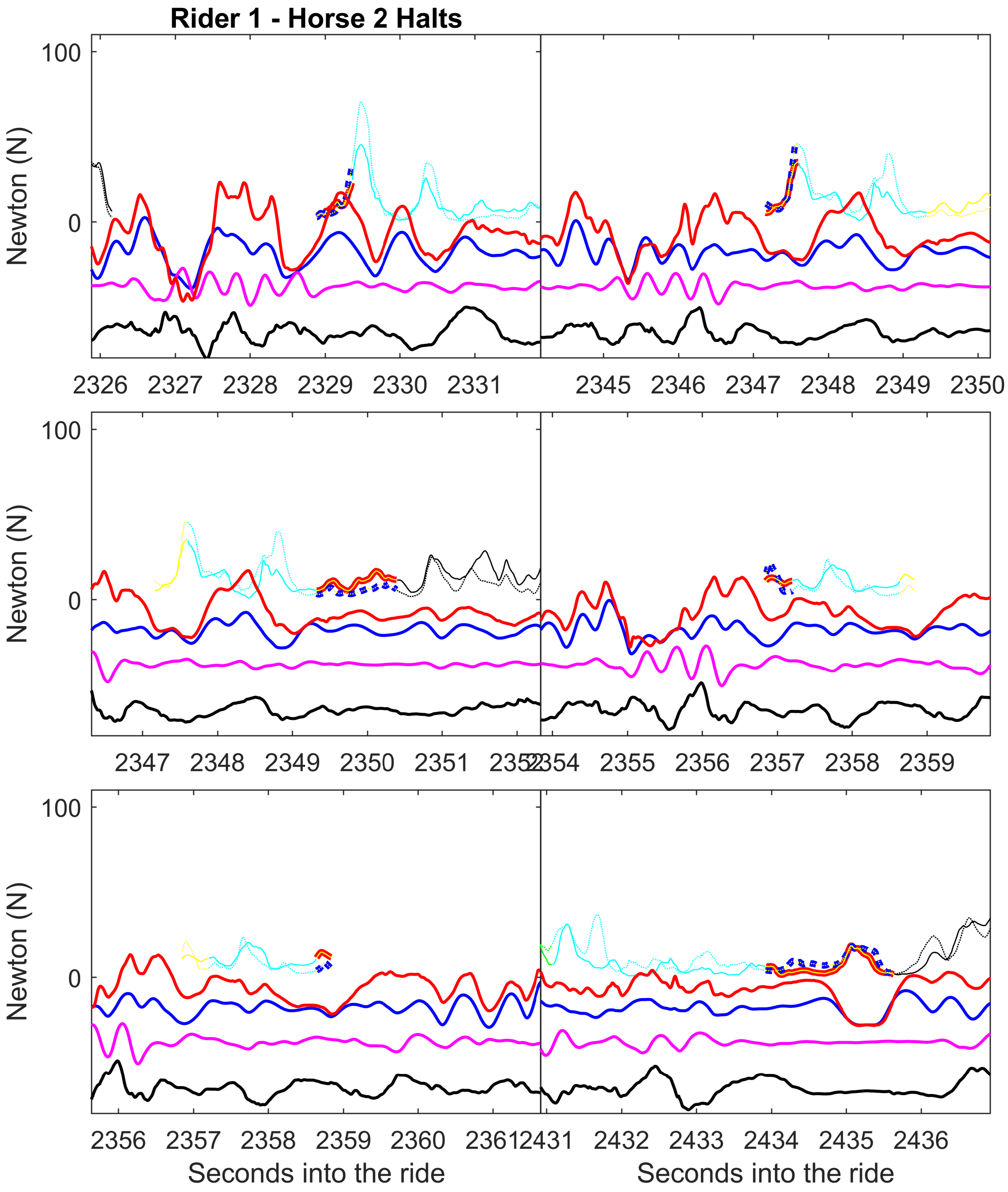

Rider 1 - Horse 2 Halts
Newton (N)
100
0
2326 2327 2328 2329 2330 2331
2345 2346 2347 2348 2349 2350
2347 2348 2349 2350 2351 2352
2354 2355 2356 2357 2358 2359
2356 2357 2358 2359 2360 2361
2431 2432 2433 2434 2435 2436
Seconds into the ride

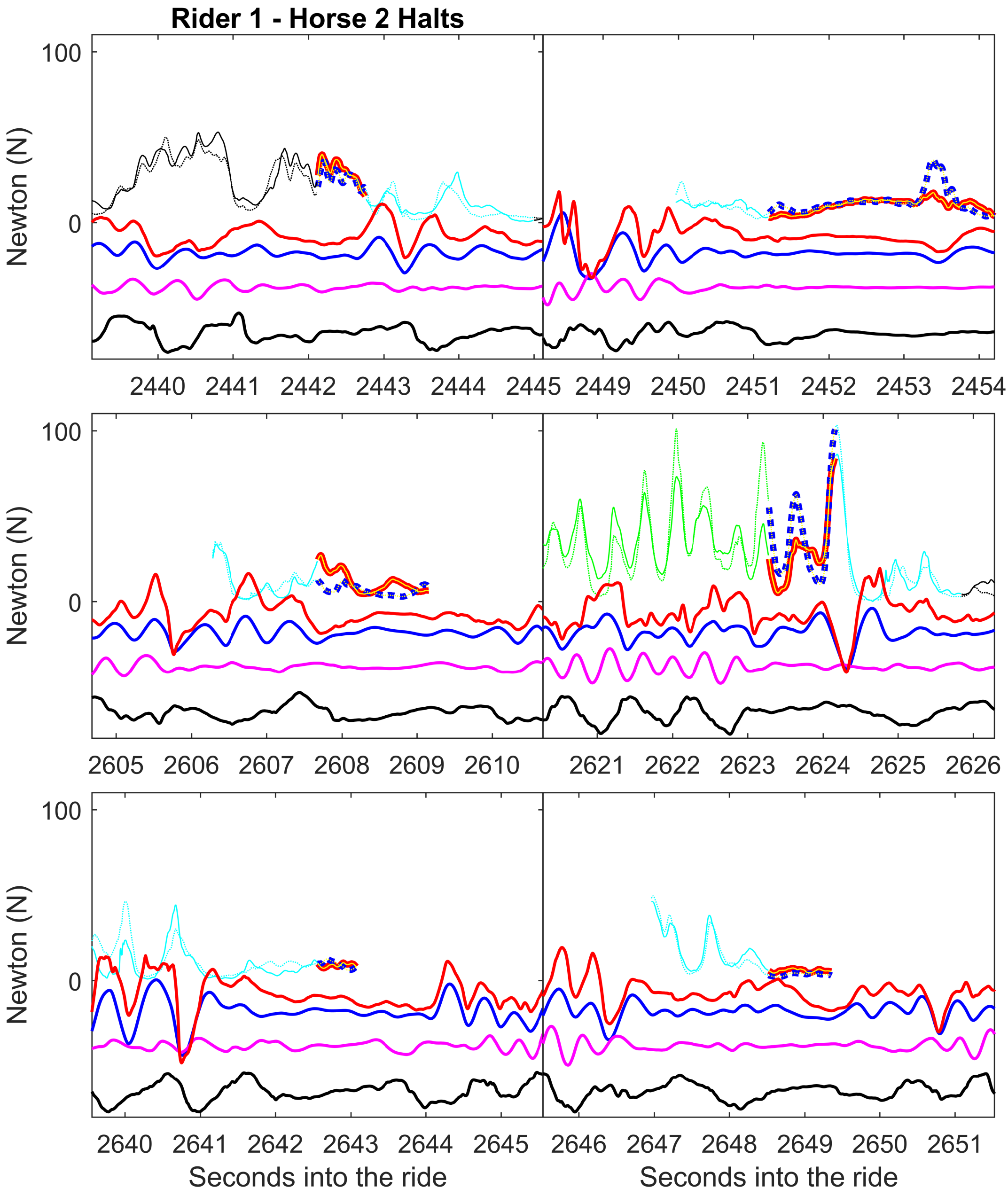

Rider 1 - Horse 2 Halts
Newton (N)
100
0
2440
2441
2442
2443
2444
2445
2449
2450
2451
2452
2453
2454
2605
2606
2607
2608
2609
2610
2621
2622
2623
2624
2625
2626
2640
2641
2642
2643
2644
2645
2646
2647
2648
2649
2650
2651
Seconds into the ride
Seconds into the ride

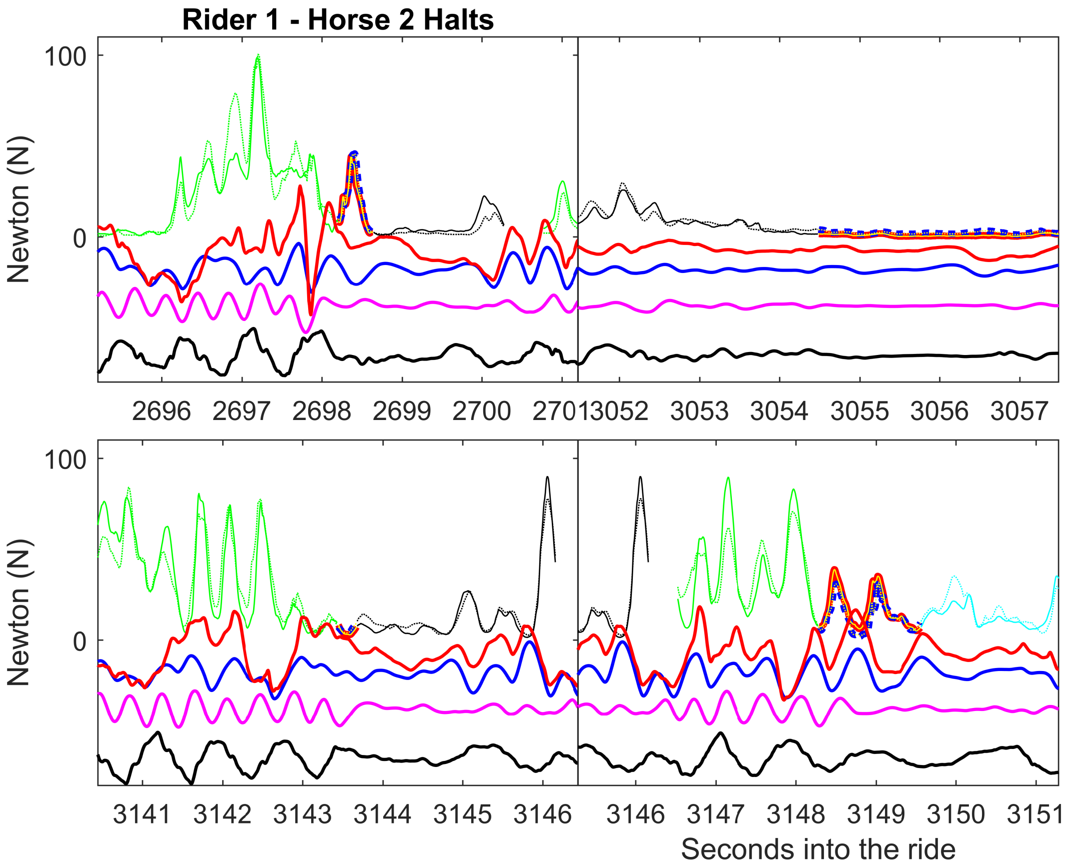

Rider 1 - Horse 2 Halts
Newton (N)
100
0
2696
2697
2698
2699
2700
2701
3052
3053
3054
3055
3056
3057
Newton (N)
100
0
3141
3142
3143
3144
3145
3146
3146
3147
3148
3149
3150
3151
Seconds into the ride

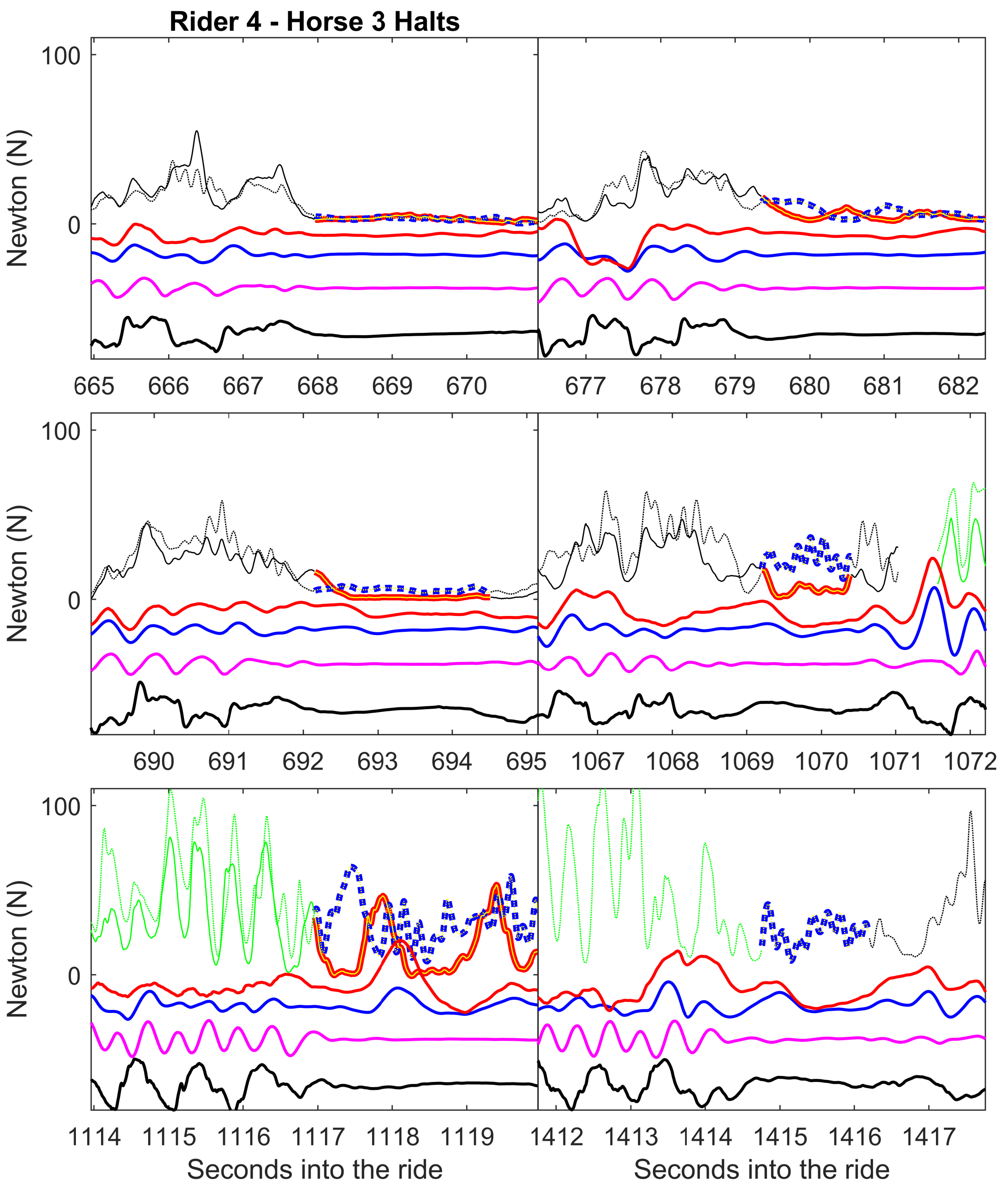

Rider 4 - Horse 3 Halts
Newton (N)
100
0
665
666
667
668
669
670
677
678
679
680
681
682
690
691
692
693
694
695
1067
1068
1069
1070
1071
1072
1114
1115
1116
1117
1118
1119
1412
1413
1414
1415
1416
1417
Seconds into the ride

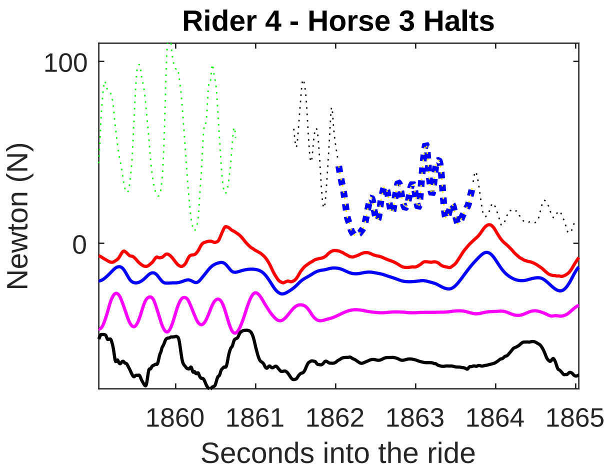

Rider 4 - Horse 3 Halts
100
0
Newton (N)
1860
1861
1862
1863
1864
1865
Seconds into the ride

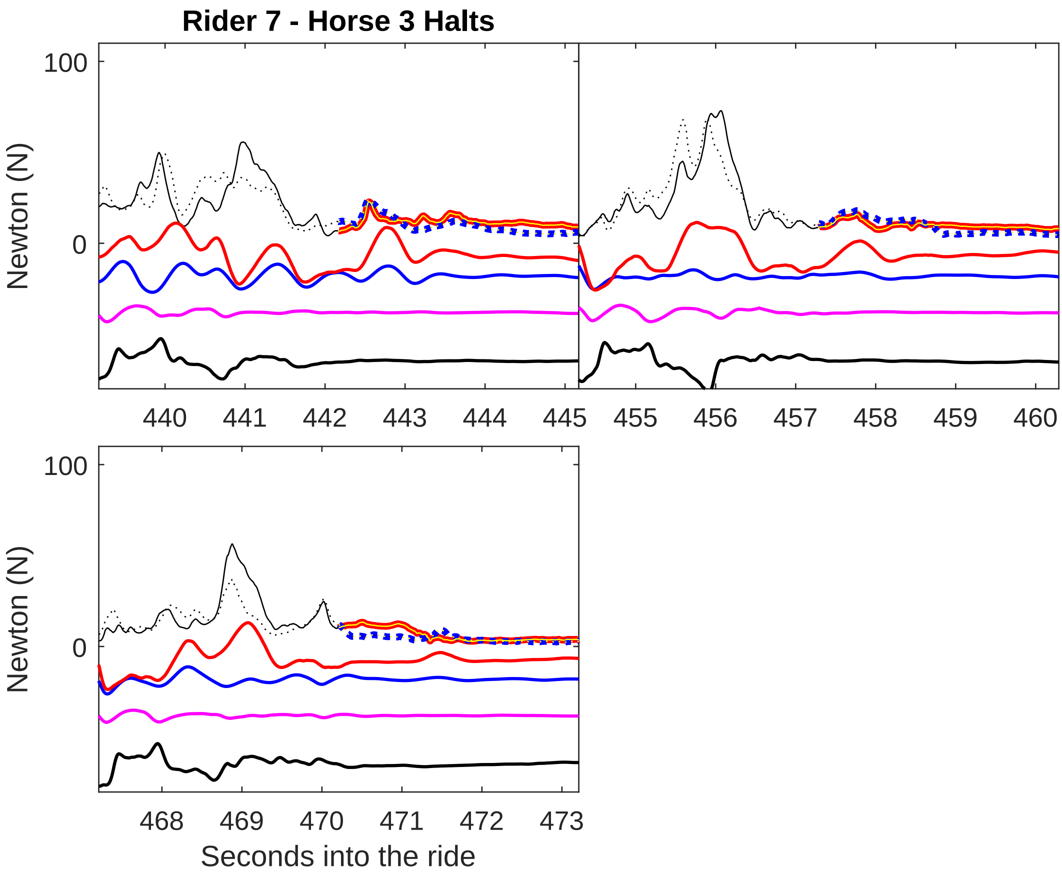

Rider 7 - Horse 3 Halts
Newton (N)
100
0
440
441
442
443
444
445
455
456
457
458
459
460
468
469
470
471
472
473
Seconds into the ride
