# Supplementary material for: Rein Tension in Transitions and Halts during Equestrian Dressage Training
Source: Animals (Basel). 2019 Sep 23;9(10):712. doi: 10.3390/ani9100712 (PMC6827353; doi:10.3390/ani9100712)
Supplement: Supplementary file 1 [file animals-09-00712-s001.zip › Table S2.pdf]

Table S2.
Definition of transition types and categories. Adapted from Argue and Clayton (1993a, b), for transitions between walk and canter only in principle.

Main types of transitions	Intermediate steps	Description
Walk-trot-type1	No	The horse springs from the diagonal support phase of the walk to the opposite diagonal through a suspension phase and begins trotting with no intermediate steps.
Walk-trot-type2	Yes	The horse breaks up two-limb support into a period of single support. Thereafter a diagonal pair is placed for a three-limb overlap. The horse lifts the limb that had been in single support and springs from the remaining diagonal into trot.
Trot-walk-type1	No	Horse proceeds directly in the walk sequence with no intermediate steps (breaking up diagonal so forelimb precedes hind limb placement).
Trot-walk-type2	Yes	The horse places a forelimb in a diagonal support, resulting in a three-limb support, but then lifts the diagonal pair leaving a front limb in single support, in place of ipsilateral support phase. The contralateral hind limb is then placed to a new diagonal from which the horse proceeds to three-limb support and begins walking.
Trot-canter-type1	No	The leading front limb is placed on the ground for a three limb overlap then proceeding through lead front limb single support to a suspension phase.
Trot-canter-type2	Yes	The horse lifts the outside (trailing to be) forelimb of a trot diagonal, resulting a non-lead single hind limb support, then places the canter diagonal, and then proceeds with placing the inside (leading) forelimb as in a normal canter stride.
Walk-canter-type1	No	Entering the diagonal support in walk, the inside hind limb is lifted and protracted more quickly to swing forward synchronously with the outside hind limb. This diagonal then becomes the first canter diagonal, followed by an inside forelimb from which the horse springs into suspension.
Walk-canter-type2	Yes	The horse transitions from walk to canter over some trot-like or undefined steps.
Canter-trot type1	No	From the canter diagonal stance phase the horse springs into suspension and begins trotting.
Canter-trot type2	Yes	Places the non-leading hind limb during lead front limb single support, resulting in a diagonal stance, after which the horse springs into a suspension phase and trots thereafter.
