# Supplementary material for: Rein Tension in Transitions and Halts during Equestrian Dressage Training
Source: Animals (Basel). 2019 Sep 23;9(10):712. doi: 10.3390/ani9100712 (PMC6827353; doi:10.3390/ani9100712)
Supplement: Supplementary file 1 [file animals-09-00712-s001.zip › Table S4.pdf]

Table S4.
Least square means estimates with standard errors (SEs) from the exploratory transition type mixed model. Outcome data are rein tension (logarithm-transformed) during the transitions analysed versus type of transition as fixed effect. Significant differences between selected comparisons are indicated by the same letter, within the same column, below the heading ‘differences’.

Outcome variable	Transition type	Est.	SE	BT	95%	CI	Differences	
Logarithm	Walk-trot-type1	2.54	0.16	12.64	(09.2	,17.3)	c	
of right	Walk-trot-type2	2.64	0.23	14.03	(08.9	,22.1)		
median rein	Trot-walk-type1	2.87	0.16	17.56	(12.9	,24.0)	c	
n=496	Trot-walk-type2	2.71	0.28	15.01	(08.7	,25.8)		
	Trot-canter-type1	3.14	0.18	23.00	(16.2	,32.7)	a	a
	Trot-canter-type2	2.79	0.20	16.29	(11.0	,24.1)	b	a
	Walk-canter-type1	2.81	0.19	16.66	(11.5	,24.2)		
	Canter-trot-type1	3.57	0.17	35.52	(25.6	,49.4)	a	
	Canter-trot-type2	3.54	0.21	34.39	(22.7	,52.1)	b	
	Canter-trot-other	3.70	0.67	40.27	(10.9	,149.4)		
Average of	Walk-trot-type1	2.54	0.13	12.72	(09.8	,16.5)	c	
logarithm of left	Walk-trot-type2	2.67	0.2	14.48	(09.8	,21.3)		
and right	Trot-walk-type1	2.86	0.13	17.41	(13.4	,22.6)	c	
median rein	Trot-walk-type2	2.75	0.27	15.64	(09.2	,26.5)		
tension n=427	Trot-canter-type1	3.10	0.16	22.14	(16.3	,30.1)	a	a
	Trot-canter-type2	2.76	0.18	15.80	(11.2	,22.3)	b	a
	Walk-canter-type1	2.72	0.16	15.19	(11.0	,20.9)		
	Canter-trot-type1	3.47	0.14	31.99	(24.2	,42.3)	a	
	Canter-trot-type2	3.56	0.18	35.21	(24.6	,50.4)	b	
	Canter-trot-other	3.56	0.59	35.17	(11.1	,111.3)		

BT- back transformed estimate
